# The Potential of Percent Agreement as an Adjunctive Diagnostic Tool for Acute Temporomandibular Disorder

**DOI:** 10.3390/jcm13185360

**Published:** 2024-09-10

**Authors:** Seo-Young Choi, Soo-Min Ok, Sung-Hee Jeong, Yong-Woo Ahn, Hye-Mi Jeon, Hye-Min Ju

**Affiliations:** 1 Department of Oral Medicine, Dental Research Institute, Pusan National University Dental Hospital, Yangsan 50612, Republic of Korea; csycsy2004@naver.com (S.-Y.C.);; 2 Department of Oral Medicine, Dental and Life Science Institute, School of Dentistry, Pusan National University, Yangsan 50612, Republic of Korea; 3 Department of Oral Medicine, Dental Clinic Center, Pusan National University Hospital, Busan 49241, Republic of Korea

**Keywords:** acute pain, chronic pain, orofacial pain, temporomandibular disorder

## Abstract

**Background/Objectives**: It is well established that individuals with chronic temporomandibular disorder (TMD) exhibit differences in their physical and psychosocial characteristics from those with acute TMD. However, few studies have analyzed the physical and psychosocial characteristics of patients with acute TMD. The objective of this cross-sectional study is twofold: first, to ascertain whether there are differences in physical and psychosocial factors among patients with acute TMD based on the percent agreement between patient-reported pain sites and pain sites identified through standardized palpation and, second, to determine the potential of percent agreement as a diagnostic and prognostic factor. **Methods**: We analyzed physical and psychosocial factors in 309 patients diagnosed with acute TMD. Of these, 171 patients were selected for an analysis of their response to treatment. These patients were divided into three groups based on their percent agreement: Group A (agreement under 80%), Group B (agreement 80–89%), and Group C (agreement 90% or over) in the initial analysis and Group a (agreement under 80%), Group b (agreement 80–89%), and Group c (agreement 90% or over) in the subsequent analysis. This study was approved by the Ethics Committee of Pusan National University Dental Hospital (IRB No. 2023-05-011, 25 May 2023). **Results**: The lower the percent agreement, the greater the parafunctional oral habits, stress, chronicity, somatization, depression, anxiety, and number of painful sites. A lower percent agreement was associated with poorer treatment outcomes. The percent agreement demonstrated a 41.2% capacity to predict residual pain after treatment. **Conclusions**: Clinicians can utilize percentage agreement as an adjunctive diagnostic tool to provide more suitable treatments to patients.

## 1. Introduction

Temporomandibular disorder (TMD) is an umbrella term encompassing temporomandibular joint (TMJ) disorders, masticatory muscle disorders, headache disorders, and disorders affecting related structures that may cause pain and functional limitations [1]. While acute pain is the body’s response to injury and is short-lived, it acts as a warning signal to protect the body from external danger; however, it can progress to chronic pain if a person is subjected to constant sensory and emotional stimulation [2,3]. Chronic pain no longer serves a protective function, and the pain itself becomes a disease that reduces quality of life [3]. Furthermore, chronic TMD pain differs from acute TMD pain in terms of its physical features, psychosocial aspects, and response to treatment, and it is classified as a distinct disease [4,5,6,7,8]. Cao et al. [5] reported that depression, anxiety, and stress levels were higher in chronic TMD patients than in acute TMD patients. Ivkovic et al. [8] observed a higher response to antidepressant treatments in patients with chronic TMD than in patients with acute TMD, indicating that the pain mechanisms in acute and chronic TMD are different. It has been reported that approximately 20% to 40% of acute TMDs can progress to chronic TMD, although the literature varies [9,10,11]. The distinction between acute and chronic pain is not absolute; however, the International Classification of Diseases (ICD-11) defines pain that persists for more than three months as chronic [4].

In accordance with the Diagnostic Criteria for Temporomandibular Disorders (DC/TMD), a diagnosis of chronic masticatory muscle disorder myofascial pain (MFP) is made when a patient describes pain as spreading or referring on the palpation of trigger points [12,13,14]. Furthermore, microtrauma, remodeling, and inflammation of the TMJ can result in pain within the joint, and, if the duration of pain is prolonged, the joint disorder may also be classified as chronic pain [15]. Given that pain is not directly visible to the examiner, the distinction between acute and chronic pain is often made solely on the basis of the patient’s self-report of their pain modalities, pain intensity, and pain duration [5,16]. Consequently, the differentiation between acute and chronic TMD becomes challenging when patients exhibit inadequate pain expression, a lack of recognition of referred pain, or difficulty in recognizing the duration of pain. This highlights the necessity of objective diagnostic tools in addition to the patient’s subjective reports of pain.

Previous studies have typically categorized patients into two groups, acute and chronic TMD, and then examined the physical and psychosocial differences between them [5,6,16,17]. Some studies have attempted to predict the prognosis of patients diagnosed with acute TMD. Epker et al. [18] published a model used to predict the likelihood of TMD chronicity based on pain intensity and the presence or absence of myofascial pain in patients with TMD symptoms. Subsequently, Dougall et al. [19] utilized this model and ascertained that patients with acute TMD who were at a higher risk of TMD chronicity were more likely to exhibit depressive and somatization symptoms than those at a lower risk. However, these studies employed the Research Diagnostic Criteria for Temporomandibular Disorders (RDC/TMD), which are not fully consistent with the currently utilized DC/TMD and assessment tools [12,20,21]. Few studies have attempted to utilize the physical and psychosocial differences observed in acute TMD patients, categorized according to DC/TMD criteria, to predict the likelihood of pain becoming chronic and the effectiveness of treatment. Recently, there have been attempts to identify acute pain that is likely to become chronic before it progresses over time [19,22]. Prior research has reported that the quality of sleep is correlated with the severity of experienced orofacial pain [23]. Additionally, a large-scale Polish study of individuals with TMD-related pain revealed a significant correlation between poor sleep quality and a diminished quality of life [24]. In light of the adverse effects on quality of life and the substantial personal and societal costs associated with TMD treatment, it is imperative to identify and mitigate the transition from acute to chronic TMD [25,26].

When the patients who visited our hospital were diagnosed according to the DC/TMD protocol, even if all of them were diagnosed with acute TMD, it was observed that the degree of awareness of painful sites varied among them. Some patients accurately reported the location of their pain, and these sites were highly concordant with those identified as painful during standardized palpation. Conversely, some patients reported pain in sites on standard palpation that were additional to those identified in their self-report. Although patients did not report referred pain on standardized palpation, the degree of agreement between the painful site on the Pain Drawing, a DC/TMD self-report instrument, and the site of pain on standardized palpation varied among patients. Although they were diagnosed with the same acute TMD, there was variability in the degree of pain reduction at their subsequent visit after oral medications, physical therapy, and behavioral modification. The present study was conducted among patients diagnosed with acute TMD. The percentage of agreement between the patient and the examiner was calculated for the purpose of quantifying the discrepancy between the painful sites identified by the patient and the examiner. The objective of this study was to ascertain whether there were significant differences among patients in terms of their physical and psychosocial factors when patients were classified according to the percent agreement between their self-reported painful sites and their painful sites on standardized palpation. Additionally, this study aimed to determine the potential of percent agreement as a diagnostic tool and predictor of prognosis. This study attempted to infer the likelihood of a patient’s acute pain progressing to chronicity by using percent agreement as an adjunct diagnostic tool to DC/TMD Axis I and II, as it is easily calculated in the clinic. Although percent agreement is easy to calculate in clinical situations, few studies have attempted to use it to diagnose patients. This study is important because it can assist clinicians in diagnosing patients and providing personalized treatment through the use of percent agreement, which is simple and easy to apply in clinical settings.

## 2. Materials and Methods

### 2.1. Data Selection

**Figure 1:**
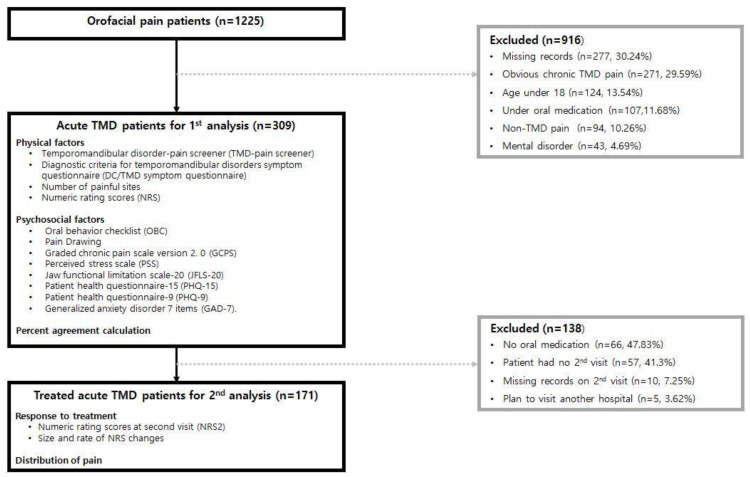
Flowchart of study’s inclusion and exclusion of patients.

Patients presenting with orofacial pain at the Department of Oral Medicine, Pusan National University Dental Hospital, between 1 January 2020 and 31 December 2022 were investigated. At their initial visit, patients were instructed to complete the self-report instruments of the DC/TMD (Axis II), a clinical assessment of the DC/TMD (Axis I), and the Perceived Stress Scale (PSS) [27,28]. All DC/TMD assessment tools (Axis I and Axis II) were used to evaluate the patients. Patients were excluded if they were under the age of 18, had an incomplete questionnaire, or had missing items in their clinical examination records (Figure 1). The exclusion criteria also included patients with neurodegenerative diseases, systemic diseases that present with pain in the orofacial region (e.g., fibromyalgia, rheumatoid arthritis, and cancer), neurovascular pain, neuropathic pain, metabolic polyneuropathy, and facial pain of dental origin. Patients taking medications that cause changes in the central nervous system that could alter pain sensation were also excluded, as were those with a history of macrotrauma to the orofacial region. Additionally, patients diagnosed with cognitive disorders, anxiety, depression, somatoform disorders, and mood disorders were excluded. Patients with active trigger points were excluded if they complained of pain with referral or pain with spreading during the clinical examination or if they complained of trigger point hypersensitivity. Patients with persistent joint pain for more than three months, which is longer than the usual healing period, were also excluded. Consequently, all patients with obvious chronic pain were excluded. A total of 309 patients (94 men and 215 women) with local myalgia, arthralgia, combined local myalgia and arthralgia, or other complaints (opening restriction, sound, discomfort, etc.) were analyzed. All patients had at least one painful site determined via self-report or standardized palpation. The clinical examination results of the 309 patients were analyzed, along with all items of the DC/TMD self-report instruments and the PSS.

To further investigate their response to treatment, 171 patients who had received oral medication and behavioral modification for at least one week and reported orofacial pain at their initial visit were further analyzed. Patients who were not prescribed oral medication (including those prescribed only topical analgesics), did not return to the clinic at the second visit, had at least one incomplete entry in their secondary record, or reported being treated at another clinic were excluded from the treatment effect analysis (Figure 1). A total of 138 patients were excluded from the secondary analysis for the following reasons: no oral medication (*n* = 66, 47.83%), no secondary visit (*n* = 57, 41.3%), missing records at secondary visit (*n* = 10, 7.25%), and scheduled visit to another hospital (*n* = 5, 3.62%). The efficacy of the treatment was evaluated using the numeric rating scale (NRS) of pain at their initial and second visits. This study was approved by the Ethics Committee of Pusan National University Dental Hospital (IRB No. 2023-05-011, approved on 25 May 2023).

### 2.2. Clinical Examination

Two or more oral and maxillofacial pain specialists performed all interviews and clinical examinations in accordance with the DC/TMD protocol. The clinical examination was conducted by employing inspection, palpation, and medical history taking. The self-reported pain intensity at the initial visit was recorded as NRS in the first analysis and NRS1 in the second analysis. Sleep duration, pain persistence, and pain-free opening were also investigated. Palpation was conducted on both patients with and without self-reported pain. The orofacial pain specialists palpated the patient’s TMJ at and around the lateral pole, bilateral superficial masseter (sM), deep masseter (dM), anterior temporalis (aT), posterior temporalis (pT), lateral pterygoid (lat pterygoid), posterior belly of the digastric (post-digastric, representing the posterior mandibular region), sternocleidomastoid (SCM), and trapezius (Tz). The palpation methods were conducted in accordance with the DC/TMD protocol [21]. The superficial masseters were palpated in three subdivisions: the origin, body, and insertion. If any of the three were painful, this was recorded as a positive result on palpation. The lateral pole of the TMJ and the surrounding area were recorded as TMJ sites. Palpation was conducted using one finger, with a force of 1 kg applied to the bilateral masseters and temporalis muscles and a force of 0.5 kg applied to the peripheral TMJ and other supplementary muscles for a minimum of two seconds, in accordance with the protocol outlined in [21]. Standardized palpation was performed bilaterally on a total of 18 zones, including the lateral TMJ, sM, dM, aT, pT, lat pterygoid, post-digastric, SCM, and Tz, and any painful sites on palpation were recorded. After the clinical examination, the number of self-reported painful sites and the number of painful sites on standardized palpation were examined.

### 2.3. Percent Agreement Calculation and Grouping

**Figure A1:**
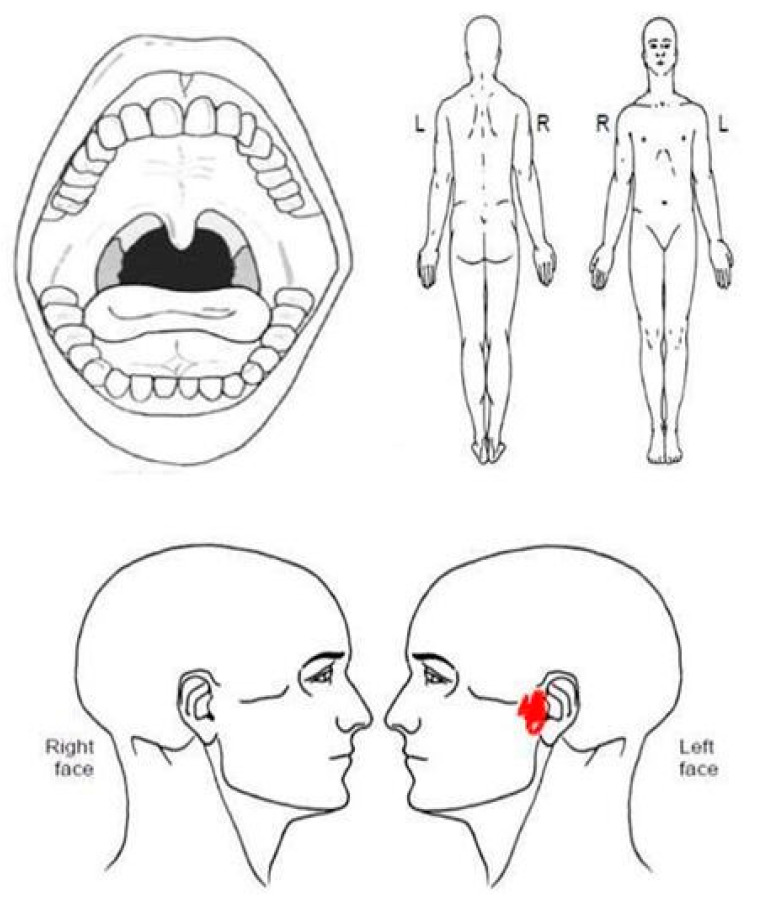
Pain Drawing Questionnaire completed by a patient. Patients were instructed to mark all sites where they felt pain, as indicated in red. Copyright International RDC/TMD Consortium Network. Available online: www.rdc-tmdinternational.org (accessed on 1 March 2024). No permission required to reproduce, translate, display, or distribute.

**Figure A2:**
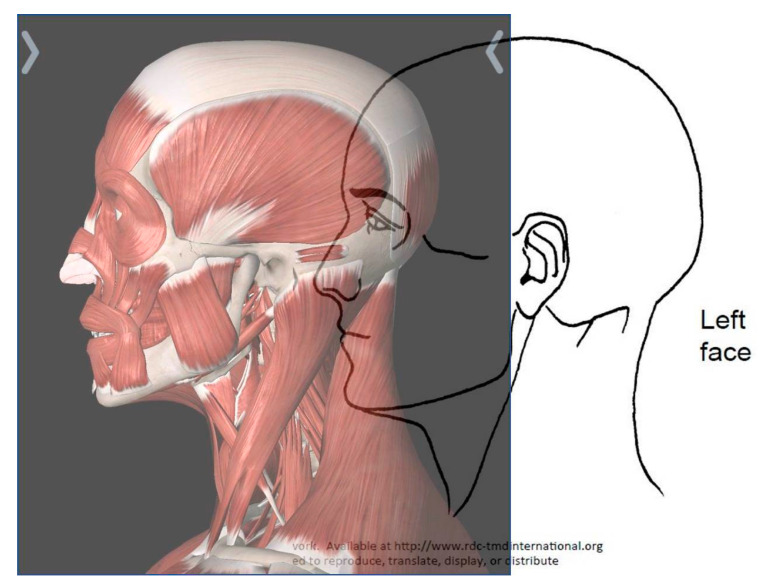
Creation of a reference figure. The figure in the Pain Drawing Questionnaire, with nothing marked on it, was superimposed on a figure featuring anatomical muscles [63]. The two figures were overlapped based on the anterior extension of the sternocleidomastoid muscle’s origin and the orbit, mandibular condyle, and lip. Copyright International RDC/TMD Consortium Network. Available online: www.rdc-tmdinternational.org (accessed on 1 March 2024). No permission required to reproduce, translate, display, or distribute.

**Figure A3:**
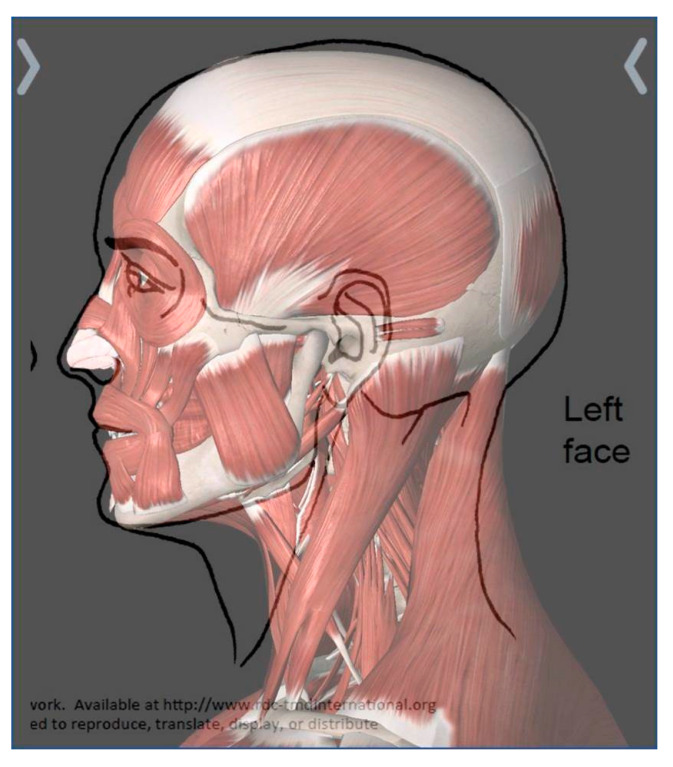
The completed reference figure. The black border lines obtained from the process shown in Figure A2 will be used to match the Pain Drawing Questionnaires filled out by patients in the future. Copyright International RDC/TMD Consortium Network. Available online: www.rdc-tmdinternational.org (accessed on 1 March 2024). No permission required to reproduce, translate, display, or distribute.

**Figure A4:**
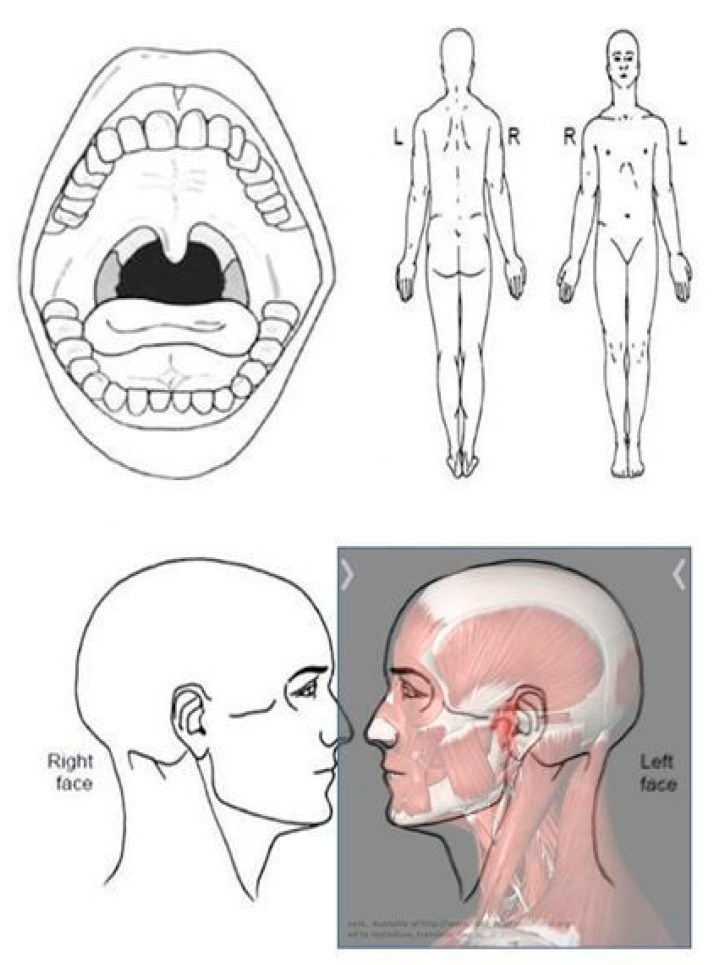
Pain Drawing Questionnaire completed by a patient with a reference figure overlapping it. Copyright International RDC/TMD Consortium Network. Available online: www.rdc-tmdinternational.org (accessed on 1 March 2024). No permission required to reproduce, translate, display, or distribute.

**Figure A5:**
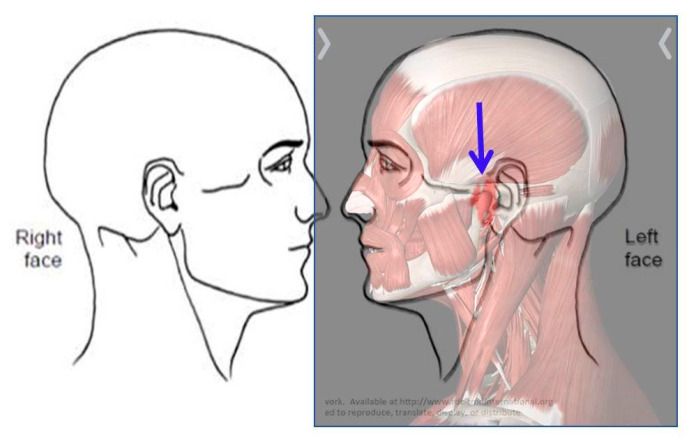
Completed figure for the interpretation of self-reported painful sites. The sites corresponding to the patient’s markings (blue arrow) in red were recorded. In this case, the patient indicated a painful site on their left TMJ. Copyright International RDC/TMD Consortium Network. Available online: www.rdc-tmdinternational.org (accessed on 1 March 2024). No permission required to reproduce, translate, display, or distribute.

The percent agreement indicates the degree to which the patient’s self-reported pain sites on their Pain Drawing match their painful sites on standardized palpation. A score of 1 was given if the patient’s self-reported pain site was the same as the standardized palpation site. If the patient and examiner disagreed on the same palpation site, a score of 0 was given. The sum of the palpation scores was divided by the total number of palpation sites (18) and multiplied by 100 to determine the percent agreement. Patient self-reported pain sites were recorded using the Pain Drawing Questionnaire. The Pain Drawing Questionnaire is a clinical assessment tool utilized to identify the location and extent of pain in patients with pain [29]. There is no established method for scoring and interpreting the Pain Drawing Questionnaire. Detailed figures of how we interpreted the Pain Drawing Questionnaire are attached in Appendix A. The patients were instructed to freely mark their painful sites with shapes, lines, arrows, etc., on the figure in the Pain Drawing Questionnaire (Figure A1). For the interpretation of the questionnaire, we created a reference figure by superimposing a Pain Drawing picture with nothing labeled onto an anatomical muscle structure picture (Figure A2 and Figure A3). On top of this reference figure, we again superimposed a picture of the Pain Drawing in which the patient freely marked the site of pain (Figure A4). We recorded the muscles corresponding to the sites marked in red by the patient (Figure A5). To avoid confusion, the patient’s statement was also used as an additional reference. Standardized palpation sites were recorded as the muscles and joints corresponding to the origin of pain that were painful when standardized palpation was performed. Patients complaining of referred pain or hypersensitivity to trigger points were excluded from the analysis, as this would indicate apparent chronic pain.

Based on the percent agreements obtained, the 309 patients were divided into 3 groups: Group A comprised those with a percent agreement of less than 80%, Group B comprised those with a percent agreement of 80–89%, and Group C comprised those with a percent agreement of 90% or greater. These grouping criteria were used in an additional study of 171 patients for a subsequent treatment effect analysis: Group a, agreement under 80%; Group b, agreement 80–89%; and Group c, agreement 90% or over.

### 2.4. Physical and Psychosocial Characteristics

Various questionnaires were used to assess the physical and psychosocial aspects of the patients. The selection of the questionnaires and the scoring and calculation of the scores followed the DC/TMD [12,30]. The following questionnaires were used: the Temporomandibular Disorder Pain Screener (TMD Pain Screener), Diagnostic Criteria for Temporomandibular Disorders Symptom Questionnaire (DC/TMD Symptom Questionnaire), Oral Behavior Checklist (OBC), Pain Drawing, Graded Chronic Pain Scale version 2.0 (GCPS), Jaw Functional Limitation Scale-20 (JFLS-20), Patient Health Questionnaire-15 (PHQ-15), Patient Health Questionnaire-9 (PHQ-9), Generalized Anxiety Disorder 7 Items (GAD-7), and Perceived Stress Scale (PSS). The assessment of TMD included both Axis I (physical diagnosis) and Axis II (psychosocial assessment). The TMD Pain Screener and DC/TMD Symptom Questionnaire correspond to the Axis I diagnosis, and the OBC, Pain Drawing, GCPS, JFLS-20, PHQ-15, PHQ-9, and GAD-7 correspond to the Axis II assessment. The Korean versions of the questionnaires were used, and the total score of each questionnaire was used in our analyses [27].

The PSS is a 10-item questionnaire that is not included in the DC/TMD self-report instruments but has been used to assess distress. A total score of less than 14 on the PSS-10 is classified as low stress, 14–26 is classified as moderate stress, and 27 or more is classified as high stress [31]. The OBC assesses parafunctional habits and is a valid risk factor for the development of TMD when its score is 25 or higher. The Pain Drawing is a questionnaire that asks patients to mark all areas of pain and is used for a comprehensive structural assessment of pain. It is also used for a differential diagnosis by asking patients to describe in detail the areas of the face and oral cavity experiencing pain in conjunction with information from the Axis I diagnosis. The GCPS version 2.0, 30-day version (GCPS), is used to determine the chronicity of pain. The characteristic pain intensity (CPI) is calculated by multiplying the mean of the GCPS items 2–4 by 10. The combination of the point of disability days and CPI is used to determine the chronic pain grade. The PHQ-15 assesses nonspecific physical symptoms, which are divided into normal, low, moderate, and high severity based on scores of 5, 10, and 15, while the PHQ-9 assesses depression, which is divided into normal, mild, moderate, moderately severe, and severe depression based on scores of 5, 10, 15, and 20. The GAD-7 measures anxiety, which is divided into normal, mild, moderate, and severe anxiety based on total scores of 5, 10, and 15.

After the completion of the questionnaires, the number of painful sites was examined to determine whether the number of painful sites reported by the patients or felt by palpation differed by the percent agreement group. The painful sites were defined as follows: In the first analysis, SRP, the number of self-reported painful sites; SPP, the number of sites of pain on palpation; and SPP-SRP, the number of sites of pain on palpation minus the number of self-reported painful sites. In the second analysis, ASRP, the number of self-reported painful sites; ASPP, the number of sites of pain on palpation; and ASPP-ASRP, the number of sites of pain on palpation minus the number of self-reported painful sites.

### 2.5. Response to Treatment

Of the 309 patients with complete questionnaires and clinical examination records, treatment effects were analyzed in the 171 patients who had been taking medication for at least one week and had an NRS of at least 1 at their first visit. The patients were divided into three groups according to their percent agreement, using the same grouping criteria as in the first analysis. In order to differentiate from the initial analysis, patients who had taken medication were classified into the three groups, as follows: Group a comprised those with a percent agreement of less than 80%, Group b comprised those with a percent agreement between 80 and 89%, and Group c comprised those with a percent agreement of 90% or greater. The number of painful sites identified through palpation and self-report was previously assessed in the initial analysis of these patients and utilized in the second analysis of their treatment response. To avoid confusion, the number of self-reported painful sites is denoted as the number of the after-treatment group’s self-reported painful sites (ASRP), and the number of painful sites on palpation is denoted as the number of the after-treatment group’s painful sites on palpation (ASPP). ASPP-ASRP is the number of painful sites on palpation minus the number of self-reported painful sites in the 171 patients at their first visit. Next, the post-treatment NRS (NRS2) was examined to determine their response to treatment. To determine the degree of pain relief, the NRS change was defined as the amount of improvement by subtracting the NRS at the second visit (NRS2) from the NRS at the first visit (NRS1). This NRS change was divided by the initial NRS (NRS1) and multiplied by 100 to define the NRS change rate. If the NRS change amount and NRS change rate were positive, the patient was considered to have improved, and if they were negative, the patient was considered to have worsened.

### 2.6. Statistical Analysis

For a comparison of the characteristics of the 3 groups, divided according to percent agreement, categorical variables (sex, duration of pain, type of medication used, and whether physical therapy was performed) were analyzed using the chi-square test to examine their distribution by percent agreement group. Other continuous variables were analyzed using a one-way ANOVA. These included age, sleep duration, pain-free opening, NRSs, number of painful sites, total questionnaire scores, and scores derived from questionnaire items. The results from each of the following instruments were recorded: the TMD Pain Screener, the DC/TMD Symptom Questionnaire, and the Pain Drawing. The mean of the total score for each of the following instruments was then analyzed by percent agreement group: the OBC, GCPS, PSS, JFLS-20, PHQ-15, PHQ-9, and GAD-7. Variables that were statistically significant in the one-way ANOVA were subjected to a further post hoc analysis. The Scheffe post hoc test was performed if the variables satisfied the equality of variances; otherwise, the Games–Howell post hoc test was performed. Variables for which a post hoc analysis was not appropriate are denoted with ‘-’. A Pearson correlation analysis was used to determine the correlation among the total scores of each questionnaire. A multiple linear regression analysis was conducted to examine the relationships between the percent agreement and treatment outcome. The distribution of pain sites in the after-treatment percent agreement group was analyzed using the chi-square test and plotted using a bar graph. All statistical analyses were performed using SPSS ver. 22.0 (IBM Corp., Armonk, NY, USA), and the minimum level of statistical significance was set at *p* < 0.05.

## 3. Results

### 3.1. Characteristics of 309 Patients for the Analysis of Their Physical and Psychosocial Aspects

The 309 patients who met the inclusion and exclusion criteria were divided into three groups according to their percent agreement. The NRS, a numerical rating of pain intensity completed at the initial visit, was higher in Group A and B, and followed by Group C (Table 1). SRP, SPP, and SPP-SRP were examined to determine whether there was a difference in the number of painful sites according to Groups A, B, and C, and a statistically significant difference was found. For all three items, Group A had the highest number, followed by Group B, and finally, the lowest was in Group C (*p* < 0.05). Other factors, such as age, sleep duration, pain-free opening, and the duration of symptoms, did not differ among the groups.

### 3.2. Psychosocial Characteristics

With a CPI of less than 50 and fewer than 3 disability points, all three groups were classified as experiencing mild chronic pain, Grade I, which corresponds to low-intensity pain without disability [32] (Table 2). Group A showed clear differences from Group C in terms of pain intensity and pain-related disability (*p* < 0.05). The CPI for the mean pain intensity over the last 30 days was significantly higher in Group A than in Group C, and the points for disability days, the points for the interference score, and the total disability points (the sum of the previous two) were also higher in Group A than in Group C. There were statistically significant differences in the OBC, JFLS-20, and PSS’s total scores according to the percent agreement group. The OBC’s total score indicates the degree of parafunctional habits, and the absolute value of the OBC’s total score was less than 25 points in all groups, but the mean score was higher in Group A than in Group C (*p* < 0.05). The total score of the JFLS-20 was higher in Group A than in the other two groups (*p* < 0.05). The PSS total score, an index of distress, was between 14 and 26 for all three groups, indicating moderate distress, but it was the lowest in Group C (*p* < 0.05). The total scores for the PHQ-15 (somatization) and PHQ-9 (depression) decreased in the order of Group A, B, and C. Group C demonstrated normal ranges (total scores of less than 5) for both questionnaires, while Groups A and B exhibited low levels of somatization and depression (*p* < 0.05). The GAD-7 total score for anxiety was within the normal range in all three groups. However, even within the normal range, the total scores of Groups A and B were higher than those of Group C (*p* < 0.05). For detailed statistical analysis results of the sub-items of the questionnaires presented in Table 2, please refer to the Supplementary Materials (Tables S1–S4).

Among the various questionnaire scores and percent agreements, the percent agreement was negatively correlated with the PHQ-15 total score (Table 3). The PHQ-15 was significantly positively correlated with the total scores of the GAD-7, PHQ-9, and PSS (*p* < 0.01). The GAD-7 total score was positively correlated with the PHQ-9 and PSS total scores (*p* < 0.01). The CPI, a measure of pain severity, was positively correlated with total disability days and functional limitations (*p* < 0.01). In particular, the correlation coefficient between the GAD-7 and PHQ-9 total scores was the highest of all analyses (*p* < 0.01).

### 3.3. Characteristics of 171 Patients Analyzed for Their Response to Treatment

Of the 309 patients previously analyzed, we examined the characteristics of 171 patients who complained of pain at their first visit and had been treated with oral medication for at least 1 week (Table 4). A total of 138 patients were included in the primary analysis but excluded from this secondary analysis. The patients included in the second analysis were divided into three groups according to their percent agreement at the time of their initial presentation: Group a (percent agreement lower than 80%), Group b (percent agreement between 80 and 89%), and Group c (percent agreement 90% or greater). There were no significant differences among the percent agreement groups in terms of age, sleep duration, amount of pain-free opening, or duration of pain (greater or less than 3 months).

The number of sites of self-reported pain in this analysis (ASRP) at the first visit was higher in Groups a and b than in Group c (*p* < 0.05). The number of sites of pain on palpation (ASPP) and the numerical difference between the ASPP and ASRP (ASPP-ASRP) were in the order of Group a, Group b, and Group c (*p* < 0.05).

We also analyzed the total score and derived items of the Axis II questionnaire for the 171 patients categorized according to their percentage of agreement in order to rule out bias due to follow-up loss (Table 5). The patients treated only with topical analgesics or only with physical therapy and behavior modification, i.e., the patients who did not receive oral medication because their pain was not severe, were not included in the analysis. Therefore, the CPI was similar in groups a, b, and c. The total scores of the other questionnaires for psychosocial factors (PSS, PHQ-15, PHQ-9, and GAD-7) were higher in the group with a lower percent agreement (*p* < 0.05). This was a statistically significant difference, similar to the previous analysis of 309 patients.

### 3.4. Response to Treatment and Pain Distribution

A total of 171 patients were treated conservatively with medication for an average of 1.67 weeks. There were no differences among the groups in terms of the time until their second visit and the duration of medication (Table 6). There was no significant difference in the type of analgesic prescribed, with the short-acting NSAIDs prescribed being zaltoprofen and ibuprofen and the long-acting NSAIDs being naproxen and celecoxib. The most commonly prescribed NSAID was zaltoprofen, in 119 of 171 patients (69.6%), followed by ibuprofen in 31 (18.1%), celecoxib in 11 (6.4%), naproxen in 6 (3.5%), and acetaminophen in 3 (1.8%). Muscle relaxants were prescribed based on the patient’s symptoms and interactions with other medications, and, again, there were no significant differences between the groups. Except for some contraindications and a lack of time, more than 90% of the patients in all three groups received physical therapy at their first visit, with no statistical differences between the groups. There was no significant difference in NRS1, the numerical pain rating at the first visit, but the NRS2, the numerical rating of pain at the second visit (NRS2), was higher in Groups a and b than in Group c (*p* < 0.05). The NRS change amount between the first and second visits was greater in Group c than in Group a (*p* < 0.05). The NRS change rate, expressed as a percentage of pain reduction based on the NRS at their first visit (NRS1), was also higher in Group c than in Groups a and b (*p* < 0.05).

**Figure 2:**
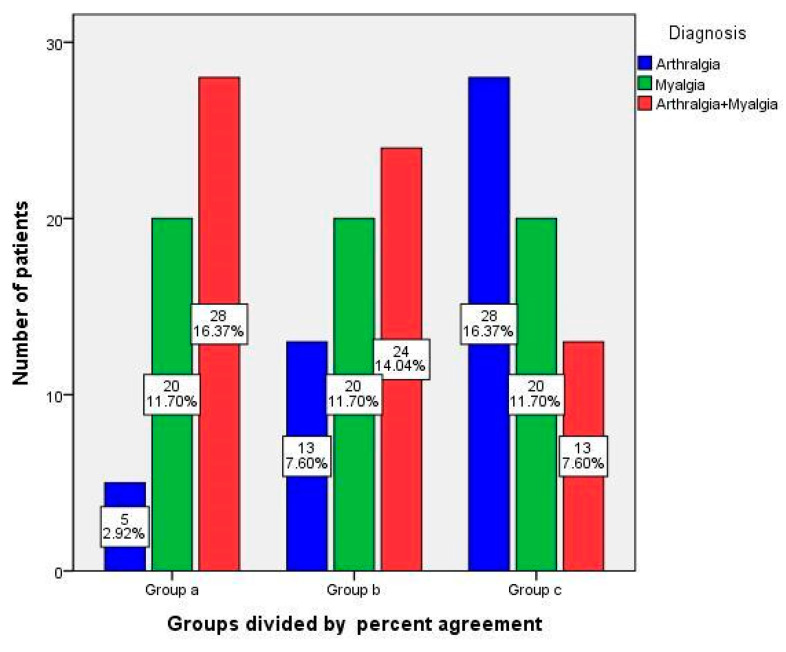
Distribution of all 171 patients by percent agreement group and by temporomandibular disorder diagnosis. Chi-square test was used (*p* = 0.000).

We examined the distribution of the patients’ painful sites according to their percent agreement group (Figure 2). Patients complaining of painful sites limited to the muscles (myalgia) were evenly distributed in all three groups. However, patients with only arthralgia were the most common in Group c, followed by Group b and Group a. Patients complaining of both arthralgia and myalgia were the most common in Group a, followed by Group b and Group c (*p* = 0.000).

### 3.5. Percent Agreement as a Predicting Factor

We performed a multiple linear regression between NSR2 and the other variables (Table 7, Figures S1 and S2). The Durbin–Watson statistic of 2.021 satisfied the independence of the residuals, and the Variance Inflation Factor (VIF) was less than 10 and close to 1, indicating that the multiple linear regression model was appropriate and without multicollinearity problems (*p* = 0.000). The explanatory power of the model was 41.2%; thus, the patient’s pain level at their second visit (NRS2) can be estimated by using the percent agreement and the NRS at their first visit (NRS1) (*p* < 0.01).

The multiple linear regression equation used to predict residual pain through the patient’s percent agreement and NR1 is as follows:

NRS2 = 2.181 + 0.677 × (NRS1) − 0.031 × (Percent agreement)

## 4. Discussion

We used percent agreement to measure the level of agreement between patients’ self-reported painful sites and sites of pain on palpation. The percent agreement is very intuitive and easy to interpret, and it is more appropriate than Cohen’s kappa values for assessing inter-observer agreement in clinical studies with an extremely low or high prevalence of abnormalities [33,34]. The substantial cut-off for percentage agreement varies in the literature, but 80% or higher is considered acceptable, and 90% or higher is considered an acceptable value if more stringent criteria are applied [35,36]. In our study, we adopted the 80% and 90% cut-offs and divided patients into three groups based on their percent agreement.

Salmos-Brito et al. [37] found that patients with chronic TMD reported a higher pain intensity than patients with acute TMD at their initial visit. In our study, the patients in Groups A and B reported a higher pain intensity at their initial presentation than the patients in Group C (Table 1). The CPI, derived from the GCPS questionnaire, which represents pain intensity, was also found to be the highest in Group A (Table 2). One study that developed a predictive model for the development of chronic TMD found the CPI to be a highly significant variable in the model [18]. Garofalo et al. [38] demonstrated that patients with a higher CPI at their initial examination were more likely to develop chronic pain. The mean CPI score for patients with acute TMD was reported to be 37.13 [38]. In our study, the mean CPI for Group A was 42.6, which was higher than the mean for acute TMD patients (Table 2). Chronic pain ratings are determined by combining the number of disability days and the CPI scores derived from the GCPS questionnaire on the chronicity of pain [30,32,39]. All three groups were classified as experiencing mild chronic pain, yet the CPI, which represents pain intensity, exhibited notable variation across the percent agreement groups. The observed differences in pain intensity, as indicated by the CPI and NRS, across groups suggest that the probability of developing chronic pain in the future may differ among groups. In addition, TMD pain intensity is associated with distress, depression, and anxiety [23].

In a study in Thailand that examined 328 TMD patients, the OBC total score was found to be significantly higher in chronic TMD patients than in acute TMD patients [16]. In our study, the OBC total score of Group A was found to be significantly higher than that of Group C (Table 2). It is known that excessive parafunctional oral behaviors can cause microtrauma to the masticatory system, which can lead to pain [40]. In a study by Louca et al. [41], patients with painful TMD exhibited significantly elevated fatigue and pain intensity in their masticatory muscles following clenching in comparison with normal individuals. Furthermore, Dawson et al. [42] reported that patients with chronic TMD exhibited elevated levels of serotonin, which was associated with increased nociceptive sensitivity, both during clenching and rest. It has been reported that prolonged masticatory muscle input and excitability can cause sensitization of the central nervous system and contribute to the development of chronic pain in TMD [43]. Based on these previous reports, it can be assumed that an increased input of peripheral primary afferent neurons through parafunctional habits is associated with the chronicity of pain and that afferent input would be greater in Group A than in the other groups.

A study by Wieckiewicz et al. [44] indicates that the PSS total score is divided into three categories: low stress (0–13), moderate stress (14–26), and high perceived stress (27–40). In our study, the PSS total scores in the three groups were between 14 and 26, corresponding to moderate stress. However, even among them, the lower the percent agreement, the higher the PSS total score (Table 2). Gameiro et al. [45] analyzed the TMJs of rats and found that morphine’s effect on nociception was reduced in stressed rats, suggesting that repetitive stress can lead to hyperalgesia. The following year, Gameiro et al. [46] proposed a vicious cycle of stress–pain–stress that may occur in patients with TMD. It has been suggested that emotional stress causes muscle hyperactivity, which modifies the endogenous opioid and serotonin systems, thereby altering the sensation of pain [46]. Altered pain sensation, in turn, causes emotional stress, entering a vicious circle again [46]. It is plausible that Groups A and B, with high levels of perceived stress, may have altered pain sensation compared with Group C, which had low total stress scores.

Elevations in one psychosocial factor were found to be significantly correlated with other psychosocial factors in our study (Table 3). A Pearson correlation coefficient greater than 0.4 is considered to indicate a moderate correlation [47]. In our study, the percent agreement was negatively correlated with the PHQ-15 total score (Table 3). The results of our study also revealed that the percent agreement is related to the number of self-reported painful sites and the number of sites of pain on palpation (Table 1). Group A exhibited the highest number of self-reported painful sites and painful sites on palpation, with a notable discrepancy between the two. This discrepancy represents the number of additional painful sites that the patients were not aware of but were identified through standardized palpation. Studies have demonstrated that, as pain becomes more chronic, patients report a higher number of painful sites [17,48]. Wilson et al. [48] demonstrated, through multiple regression, that the number of extraoral sites of pain on palpation were strongly related to somatization. Their study found that the higher the number of sites of pain on palpation felt by the patient, the greater the degree of somatization [48]. Nickel et al. [49] proposed that the PHQ-15 total score may be indicative of abnormalities in sensory processing and could serve as a potential clinical marker of nociplastic chronic pain. In other words, a lower percent agreement and a greater number of sites where the patient feels pain on palpation are associated with more somatization, which, in turn, is associated with chronic pain. Additionally, in our study, the PHQ-15 total score was positively correlated with the PHQ-9 total score (Table 3). The PHQ-15 and PHQ-9 results showed that Group C was within the normal range, while Groups A and B exhibited scores indicating low severity (Table 2) [30].

We confirmed that the PHQ-15 and PHQ-9 total scores statistically significantly decreased in the order of Group A, Group B, and Group C. This tendency is similar to the results of a comparative analysis of patients with myalgia, MFP, and fibromyalgia, in which their PHQ-15 and PHQ-9 total scores increased with an increasing chronicity of pain [6]. Furthermore, the total scores of the PHQ-15 and PHQ-9 in Group A were higher than the mean of MFP patients reported by Winocur-Arias et al. [50]. The total scores of the PHQ-15 and PHQ-9 were previously shown to be important factors in the progression to chronic TMD [51]. The total score of GAD-7 was the lowest in Group C (Table 2). In a study by Barjandi et al. [6], the mean GAD-7 total score of myalgia patients was reported to be 3.0, while the mean GAD-7 total score of MFP patients was reported to be 7.0. In our study, the GAD-7 total scores of Groups A and B were found to be significantly higher than those of Group C, and they were also higher than those of the myalgia patients in the Barjandi et al. study [6]. The correlation coefficient between the PHQ-9 and GAD-7 total scores in our study was 0.786, indicating a clear positive correlation (Table 3). Other previous studies have also reported correlations between PHQ-9 and GAD-7 total scores in patients with TMD [44,52]. Studies employing a different instrument, the Symptom Checklist-90-Revised, have also consistently reported elevated levels of depression and anxiety in patients with TMD [7,53].

A number of studies have demonstrated that the onset and persistence of TMD are strongly associated with a number of psychosocial factors [7,17,18,19,53,54]. In a 3-year prospective cohort study, Slade et al. [54] noted that depression was a significant predictor of TMD development. A larger prospective cohort study demonstrated that the odds ratio of TMD development was significantly associated with psychosocial factors, including anxiety, somatization, and depression [53]. Varun et al. [17] compared healthy controls, myalgia, and MFP patients and found that MFP patients reported significantly higher anxiety, depression, and nonspecific physical symptoms than myalgia patients. These psychological factors (e.g., depression, stress, somatization, and anxiety) may contribute to the persistence of TMD through an intermediate phenotype of high psychological distress, which leads to the chronicity of pain [55,56].

In the second analysis, conducted to determine the response to conservative treatment, the NRS2 of Groups a and b was higher than that of Group c, indicating that the degree of residual pain after treatment was different among the groups (Table 6). Nonsteroidal anti-inflammatory drugs (NSAIDs) were recommended as the first-line treatment for TMD by Dym et al. [57]. However, the analgesic effect of NSAIDs is not well established in chronic TMD [58]. Dionne et al. [59] suggested that the use of NSAIDs in acute facial pain is useful, but there is a limited evidence base for the repeated administration of NSAIDs in chronic orofacial pain. Furthermore, a previous study found that chronic TMD pain responds better to antidepressant treatment than acute TMD pain [8]. In our study, we observed that Group a was less responsive to medication than Group c. Their disparate treatment responses indicate that the pain mechanisms in the two groups may not be identical, despite their diagnosis of the same acute TMD. The distribution of myalgia and arthralgia differed according to the percent agreement group (Figure 2). Patients complaining of both joint and muscle pain were the most prevalent in the low percent agreement group (Group a), and they exhibited high somatization and depression scores (Table 5). Dougall et al. [19] divided patients with acute TMD into three groups, namely, muscle pain disorder (MPD), disc displacement (DD), and degenerative joint disorder (DJD), and examined the psychosocial factors associated with these combinations. The results indicated that patients with MPD and comorbidities reported greater pain and higher somatization and depression scores than patients with either diagnosis alone [19]. Garofalo et al. [38] also reported that masticatory muscle disorders and joint problems, unlike disc displacements, have a higher rate of chronicity. Furquim et al. [60] reported that an increase in nociceptive input in inflamed joints can induce the central sensitization of surrounding masticatory muscles. Varun et al. [17] found that the number of sites of pain on palpation increases as musculoskeletal pain becomes chronic. In light of the aforementioned studies and the high prevalence of multiple diagnoses and elevated depression and somatization scores in Group a, it is somewhat implausible to consider Group a as a straightforward case of acute TMD.

The second analysis in this study examined the feasibility of using the self-reported pain value at the initial visit and the calculated percent agreement from palpation as predictors of how much pain the patient will be in at their next visit (Table 7). In addition to estimating the tendency of pain to progress to chronic in individuals diagnosed with the same acute pain, we found that their percent agreement can also provide a rough prediction of the pain intensity that will remain after future medication. Litt et al. [61] reported that psychosocial factors may decrease the response to treatment in patients with TMD, which indicates the necessity of subgrouping individuals by specific pain disorders, using both psychosocial and functional features, for appropriate treatment [62].

When considered collectively, individuals diagnosed with acute pain and exhibiting a low percent agreement tend to exhibit less common outcomes on assessments of their physical and psychosocial factors. Patients with a low percent agreement report a higher pain intensity and engage in more parafunctional habits that may increase their afferent sensory input than patients with a high percent agreement. It is also possible that their pain inhibitory system is less functional due to stress. Patients with a low percent agreement tend to report more painful sites when palpated than those with a high percent agreement, suggesting a possible increase in somatization. Increased somatization is often accompanied by depression and anxiety, and these psychosocial factors increase the probability that a patient’s pain will become chronic in the future. The inferior response to medication in the low percent agreement group, coupled with the higher prevalence of patients with multiple diagnoses, suggests that they may be on an intermediate pathway from acute pain to chronic pain. Chronic TMD does not respond well to conservative TMD treatments, causing patients to suffer from psychiatric comorbidities and increasing socioeconomic costs [26,61]. This study suggests that, even among patients diagnosed with acute TMD, there are differences in the likelihood of chronicity and the progression of pain. Consequently, the need for further classification of acute TMD is proposed. A simple, clinically available percentage agreement is suggested as a means of predicting a patient’s response to treatment and providing more aggressive and appropriate treatments to prevent the chronicity of TMD and improve patient quality of life.

This study is limited by the fact that the diagnosis was not made by a single examiner, and the sample size was relatively small; additionally, the cross-sectional nature of the study makes it difficult to analyze long-term outcomes. To confirm the conclusions presented in this study, it will be necessary to conduct longer-term, longitudinal, systematic studies with larger populations.

## 5. Conclusions

It is important to assess the likelihood and extent of progression toward chronic TMD in patients diagnosed with acute TMD, as it can help to provide the most appropriate treatment plan for each individual. Percent agreement is a physical factor that allows clinicians to easily estimate the somatization of pain and predict residual pain after initial treatments. Rather than dividing acute and chronic pain by 3 months, we suggest that it is more appropriate to use a continuous variable, percent agreement, to reflect the progression of the chronicity of pain. When patients diagnosed with acute TMD were sub-grouped using percent agreements, a lower percent agreement was associated with more parafunctional oral habits, more perceived stress, more intense pain, more physical symptoms, more depression, more anxiety, more painful sites, and less responsiveness to treatment. This suggests that, even among acute TMD patients, some patients may be in the midst of becoming chronic, thus calling for a further stratification of patients using DC/TMD Axes I and II. In addition, the clinical factor of the percent agreement has potential as a prognostic factor for medication. Continued efforts are needed to predict the likelihood of chronicity in patients with acute TMD using a variety of methods in order to improve patient quality of life.

## Figures and Tables

**Table 1:** Characteristics of all 309 patients in terms of physical and psychological aspects.

Characteristics		Mean (±SD)			*p*-Value	Scheffe/Games–Howell †
		Group A (*n* = 70)	Group B (*n* = 108)	Group C (*n* = 131)		
Sex	Male (%)	19 (27.1)	33 (30.6)	42 (32.1)	0.770	-
	Female (%)	51 (72.9)	75 (69.4)	89 (67.9)		
Age		38.93 (±15.90)	40.53 (±17.28)	41.78 (±19.15)	0.554	-
Sleep duration (h/day)		6.58 (±1.83)	6.36 (±1.39)	6.78 (±1.37)	0.096	-
Pain-free opening (mm)		35.19 (±10.33)	37.01 (±10.25)	36.15 (±9.47)	0.470	-
Duration of initial symptoms (mon)		36.66 (±54.06)	29.22 (±51.06)	20.63 (±50.20)	0.102	-
NRS		4.50 (±2.57)	4.41 (±2.47)	3.48 (±2.43)	0.004 *	A, B > C
SRP		3.66 (±2.65)	2.38 (±2.09)	1.47 (±2.05)	0.000 *	A > B > C †
SPP		6.26 (±3.52)	3.00 (±2.36)	1.61 (±2.13)	0.000 *	A > B > C †
SPP-SRP		2.63 (±3.66)	0.64 (±1.74)	0.14 (±0.87)	0.000 *	A > B > C †

Group A, percent agreement less than 90%; Group B, percent agreement 80–89%; Group C, percent agreement 90% or more; A, B, and C, Groups A, B, and C; NRS, numeric rating scale at first visit; SRP; number of self-reported painful sites; SPP, number of sites of pain on palpation; SPP-SRP, number of sites of pain on palpation minus number of self-reported painful sites. A positive SPP-SRP indicates that the patient felt pain in more sites on palpation than they reported. * *p* < 0.05; † analyzed using Games–Howell post hoc test.

**Table 2:** Scores and derivatives for psychosocial questionnaires of 309 patients.

	Mean (±SD)			*p*-Value	Scheffe/Games–Howell †
	Group A (*n* = 70)	Group B (*n* = 108)	Group C (*n* = 131)		
CPI	42.6 (±27.66)	36.56 (±27.14)	28.6 (±24.23)	0.001 *	A > C
Points for disability days	2.23 (±1.19)	1.94 (±1.35)	1.62 (±1.42)	0.006 *	A > C †
Points for interference score	0.90 (±1.08)	0.62 (±1.04)	0.38 (±0.80)	0.001 *	A > C †
Total disability points	3.13 (±1.91)	2.56 (±1.97)	2.0 (±1.85)	0.000 *	A > C
OBC total score	21.24 (±11.22)	18.44 (±8.05)	17.58 (±9.90)	0.036 *	A > C
JFLS-20 total score	52.34 (±37.67)	40.11 (±31.53)	38.40 (±30.95)	0.012 *	A > B, C
PSS total score	18.66 (±6.77)	18.10 (±6.58)	15.10 (±5.72)	0.000 *	A, B > C
PHQ-15 total score	8.71 (±4.96)	6.26 (±4.01)	4.05 (±3.60)	0.000 *	A > B > C †
PHQ-9 total score	7.77 (±5.71)	5.39 (±4.89)	3.69 (±3.92)	0.000 *	A > B > C †
GAD-7 total score	4.87 (±4.45)	3.61 (±3.79)	2.30 (±2.89)	0.000 *	A, B > C †

Group A, percent agreement less than 90%; Group B, percent agreement 80–89%; Group C, percent agreement 90% or more; A, B, and C, Groups A, B, and C; * *p* < 0.05; † analyzed using Games–Howell post hoc test.

**Table 3:** Pearson correlation analysis of questionnaire results and percent agreement.

	Percent Agreement	GAD Total Score	PHQ-9 Total Score	PHQ-15 Total Score	PSS Total Score	JFLS Total Score	CPI	Total Disability Points
Percent agreement	1							
GAD-7 total score	−0.327 *	1						
PHQ-9 total score	−0.377 *	0.786 *	1					
PHQ-15 total score	−0.415 *	0.562 *	0.629 *	1				
PSS total score	−0.252 *	0.619 *	0.590 *	0.436 *	1			
JFLS-20 total score	−0.172 *	0.236 *	0.321 *	0.330 *	0.229 *	1		
CPI	−0.190 *	0.215 *	0.258 *	0.313 *	0.252 *	0.502 *	1	
Total disability points	−0.197 *	0.198 *	0.254 *	0.269 *	0.283 *	0.498 *	0.760 *	1

* *p* < 0.01.

**Table 4:** Characteristics of a total of 171 patients further analyzed for their response to treatment.

		Mean (±SD)			*p*-Value	Games–Howell
		Group a (*n* = 53)	Group b (*n* = 57)	Group c (*n* = 61)		
Sex	Male (%)	13 (24.5)	15 (26.3)	19 (31.3)	0.711	-
	Female (%)	40 (75.5)	42 (73.7)	42 (68.9)		
Age		41.57 (±17.60)	41.82 (±17.66)	45.87 (±20.70)	0.382	-
Sleep duration		6.34 (±1.80)	6.35 (±1.46)	6.77 (±1.80)	0.293	-
Pain-free opening		33.50 (±9.14)	34.30 (±9.95)	32.89 (±8.49	0.706	-
Duration of pain	Under 3 mon (%)	19 (35.8)	20 (52.6)	32 (52.5)	0.129	-
	Over 3 mon (%)	34 (64.2)	27 (47.4)	29 (47.5)		
ASRP		3.40 (±2.31)	2.65 (±1.96)	1.56 (±1.28)	0.000 *	a, b > c
ASPP		6.30 (±3.09)	3.54 (±2.32)	1.62 (±1.29)	0.000 *	a > b > c
ASPP-ASRP		2.94 (±3.08)	0.93 (±1.65)	0.07 (±0.70)	0.000 *	a > b > c

Group a, percent agreement less than 90%; Group b, percent agreement 80–89%; Group c, percent agreement 90% or more; a, b, and c, Groups a, b, and c; ASRP; after-treatment group number of self-reported painful sites; ASPP, after-treatment group number of sites of pain on palpation in these groups; ASPP-ASRP, number of sites of pain on palpation minus number of self-reported painful sites in after-treatment group. A positive ASPP-ASRP indicates that the patient felt pain in more sites on palpation than they reported at their first visit. * *p* < 0.05.

**Table 5:** Scores and derivatives for psychosocial questionnaires of 171 patients.

	Mean (±SD)			*p*-Value	Scheffe/Games–Howell †
	Group a (*n* = 55)	Group b (*n* = 55)	Group c (*n* = 61)		
CPI	49.6 (±27.71)	43.97 (±28.07)	40.16 (±22.06)	0.154	-
Points for disability days	2.31 (±1.18)	2.27 (±1.20)	2.23 (±1.22)	0.938	-
Points for interference score	0.89 (±1.12)	0.76 (±1.17)	0.57 (±0.30)	0.272	-
Total disability points	3.20 (±1.91)	3.03 (±1.90)	2.80 (±1.64)	0.497	-
OBC total score	22.27 (±11.61)	18.55 (±8.24)	18.12 (±10.32)	0.061	-
JFLS-20 total score	59.52 (±40.00)	45.27 (±37.49)	52.60 (±30.01)	0.115	-
PSS total score	19.26 (±6.29)	17.47 (±6.42)	16.28 (±5.68)	0.034 *	a > c
PHQ-15 total score	9.02 (±4.88)	6.25 (±4.12)	4.66 (±4.00)	0.000 *	a > b, c
PHQ-9 total score	8.145 (±6.31)	5.22 (±5.08)	4.61 (±4.43)	0.001 *	a > b, c †
GAD-7 total score	4.98 (±4.54)	3.09 (±3.16)	2.92 (±3.58)	0.007 *	a > b, c

Group a, percent agreement less than 90%; Group b, percent agreement 80–89%; Group c, percent agreement 90% or more; a, b, and c, Groups a, b, and c; * *p* < 0.05; † analyzed using Games–Howell post hoc test.

**Table 6:** Duration and methodology of treatment and the effectiveness of the treatment in 171 patients.

		Mean (±SD)			*p*-Value	Scheffe
		Group a (*n* = 53)	Group b (*n* = 57)	Group c (*n* = 61)		
Duration until second visit (days)		27.38 (±25.15)	23.32 (±13.45)	24.57 (±17.13)	0.521	-
Duration of medication (wks)		1.70 (±0.57)	1.66 (±0.63)	1.66 (±0.57)	0.930	-
Analgesics	Short-acting NSAIDs (%)	48 (90.6)	47 (82.5)	56 (91.8)	0.218	-
	Long-acting NSAIDs (%)	3 (5.7)	9 (15.8)	5 (8.2)		
	Acetaminophen	2 (3.8)	1 (1.8)	0 (0)		
Muscle relaxant	Yes	42 (79.2)	53 (93.0)	55 (90.2)	0.069	-
	No	11 (20.8)	4 (7.0)	6 (9.8)		
Physical therapy	Yes	51 (98.1)	55 (96.5)	56 (91.8)	0.254	-
	No	1 (1.9)	2 (3.5)	5 (8.2)		
NRS1		5.37 (±1.89)	5.19 (±1.98)	4.66 (±1.93)	0.127	-
NRS2		3.59 (±2.22)	3.25 (±2.26)	2.04 (±1.87)	0.000 *	a, b > c
NRS change amount		1.77 (±1.63)	1.94 (±1.87)	2.62 (±1.85)	0.027 *	a < c
NRS change rate (%)		34.93 (±30.16)	38.06 (±34.99)	58.80 (±33.27)	0.000 *	a, b < c

Group a, percent agreement less than 90%; Group b, percent agreement 80–89%; Group c, percent agreement 90% or more; a, b, and c, Groups a, b, and c; short-acting NSAIDs include zaltoprofen and ibuprofen, and long-acting NSAIDs include naproxen and celecoxib; NRS1, numeric rating sale at first visit; NRS2, numeric rating sale at second visit. * *p* < 0.05.

**Table 7:** Multiple regression of NRS2 with NRS1 and percent agreement.

	B	SE	β	t	*p*-Value	TOL	VIF
(Constant)	2.181	1.020		2.139	0.034		
NRS1	0.677	0.068	0.596	10.006	0.000 *	0.976	1.024
Percent agreement	−0.031	0.011	−0.177	−2.972	0.003 *	0.976	1.024
F (*p*)	60.467 (0.000 *)						
adjR^2^	0.412						
Durbin–Watson	2.021						

* *p* < 0.01.

## Data Availability

The raw data supporting the conclusions of this article will be made available by the authors on request, without undue reservation.

## Supplementary Materials

The following supporting information can be downloaded at https://www.mdpi.com/article/10.3390/jcm13185360/s1, Table S1: GCPS and GCPS derivatives for pain analysis in 309 patients; Table S2: OBC, JFLS, and PSS total scores and items that differ significantly by percent agreement group in 309 patients; Table S3: Nonspecific somatic symptom scores reported via PHQ-15 in 309 patients; Table S4: Depression, measured with PHQ-9, and anxiety, measured with GAD-7, scores in 309 patients; Figure S1: ZRESID normal P-P plot of multiple linear regression analysis; Figure S2: Scatterplot of ZRESID by ZPRED of multiple linear regression analysis.
