# Supplementary material for: The Potential of Percent Agreement as an Adjunctive Diagnostic Tool for Acute Temporomandibular Disorder
Source: J Clin Med. 2024 Sep 10;13(18):5360. doi: 10.3390/jcm13185360 (PMC11432075; doi:10.3390/jcm13185360)
Supplement: Supplementary file 1 [file jcm-13-05360-s001.zip › jcm-3193980-supplementary.pdf]

Table S1. GCPS and GCPS derivatives for pain analysis in 309 patients.

	Mean (±SD)			*p*-value	Scheffe/ Games-Howell†
	Group A (n=70)	Group B (n=108)	Group C (n=131)		
GCPS-2	3.90 (±2.95)	3.16 (±2.86)	2.47 (±2.42)	0.002*	A>C
GCPS-3	5.23 (±3.18)	4.53 (±3.21)	3.60 (±2.96)	0.001*	A>C
GCPS-4	3.86 (±2.81)	3.3 (±2.69)	2.51 (±2.34)	0.001*	A>C
GCPS-6	3.29 (±2.84)	2.68 (±2.69)	1.91 (±2.33)	0.001*	A>C
GCPS-7	3.23 (±2.95)	2.12 (±2.68)	1.56 (±2.37)	0.000*	A>B,C†
GCPS-8	3.01(±2.80)	2.07 (±2.71)	1.35 (±2.58)	0.000*	A>C†
CPI	42.6 (±27.66)	36.56 (±27.14)	28.6 (±24.23)	0.001*	A>C
Interference score	31.76 (±26.87)	22.84 (25.63)	16.08 (±20.90)	0.000*	A>C†
Point for interference score	0.90 (±1.08)	0.62 (±1.04)	0.38 (±0.80)	0.001*	A>C†
Point for disability days	2.23 (±1.19)	1.94 (±1.35)	1.62 (±1.42)	0.006*	A>C†
Total disability point	3.13 (±1.91)	2.56 (1.97)	2.0 (±1.85)	0.000*	A>C

A,B and C, for Groups **A**,**B** and **C**; CPI, characteristic pain intensity. The number after the questionnaire name indicates the number of the individual question in that questionnaire. **p*<0.05, †Analyzed by Games-Howell post hoc.

Table S2. OBC, JFLS, and PSS total scores and which of these items differ significantly by percent agreement group in 309 patients.

	Mean (±SD)			*p*-value	Scheffe/ Games-Howell†
	Group A (n=70)	Group B (n=108)	Group C (n=131)		
OBC total score	21.24 (±11.22)	18.44 (±8.05)	17.58 (±9.90)	0.036*	A>C
OBC-1	1.91 (±1.69)	1.08 (±1.50)	1.05 (±1.48)	0.001*	A>B,C†
OBC-5	1.11 (±1.20)	0.76 (±1.07)	0.69 (±1.11)	0.034*	A>C†
OBC-6	1.53 (±1.38)	0.74 (±0.96)	0.60 (±1.01)	0.000*	A>B,C†
JFLS-20 total score	52.34 (±37.67)	40.11 (±31.53)	38.40 (±30.95)	0.012*	A>B,C
JFLS-20-1	6.36 (±3.43)	5.40 (±3.52)	4.88 (±3.70)	0.021*	A>C
JFLS-20-9	3.04 (±3.29)	1.93 (±2.64)	2.01 (±2.59)	0.018*	A>B,C†
JFLS-20 mastication	3.41 (±2.30)	3.02 (±2.34)	2.80 (±2.38)	0.212	-
JFLS-20 mobility	3.74 (±2.70)	2.97 (±2.51)	2.97 (±2.33)	0.075	-
PSS total score	18.66 (±6.77)	18.10 (±6.58)	15.10 (±5.72)	0.000*	A,B>C
PSS-1	1.47 (±1.11)	1.48 (±1.14)	1.06 (±0.99)	0.004*	A,B>C
PSS-2	1.43 (±1.14)	1.28 (±1.20)	0.87 (±1.03)	0.000*	A,B>C†
PSS-3	2.24 (±1.12)	2.13 (±1.16)	1.62 (±1.20)	0.000*	A,B>C
PSS-6	1.60 (±1.07)	1.31 (±0.97)	1.15 (±0.92)	0.007*	A>C

A,B and C, for Groups A,B and C; JFLS-20 mastication, mean of answers for questions 1-6, JFLS-20 mobility, mean of answers for questions 7-10. The number after the questionnaire name indicates the number of the individual question in that questionnaire. **p*<0.05, †Analyzed by Games-Howell post hoc.

Table **S3**. Nonspecific somatic symptom scores reported via PHQ-15 in 309 patients.

	Mean (±SD)			*p*-value	Scheffe/ Games-Howell†
	Group A (n=70)	Group B (n=108)	Group C (n=131)		
PHQ-15 total score	8.71 (±4.96)	6.26 (±4.01)	4.05 (±3.60)	0.000*	A>B>C†
PHQ-15-1	0.50 (±0.65)	0.31 (±0.56)	0.15 (±0.38)	0.000*	A>B,C†
PHQ-15-2	0.93 (±0.67)	0.72 (±0.67)	0.50 (±0.65)	0.000*	A>C
PHQ-15-3	0.80 (±0.67)	0.63 (±0.70)	0.50 (±0.67)	0.011*	A>C
PHQ-15-4 (female)	0.46 (±0.70)	0.51 (±0.70)	0.27 (±0.54)	0.018*	B>C†
PHQ-15-5	1.01 (±0.79)	0.76 (±0.72)	0.48 (±0.65)	0.000*	A>B>C
PHQ-15-6	0.41 (±0.58)	0.17 (±0.42)	0.07 (±0.28)	0.000*	A>B,C†
PHQ-15-7	0.69 (±0.67)	0.41 (±0.58)	0.27 (±0.49)	0.000*	A>B,C†
PHQ-15-9	0.46 (±0.67)	0.23 (±0.47)	0.09 (±0.29)	0.000*	A>B>C†
PHQ-15-10	0.23 (±0.48)	0.09 (±0.29)	0.03 (±0.17)	0.002*	A>C†
PHQ-15-11	0.54 (±0.65)	0.44 (±0.60)	0.26 (±0.52)	0.003*	A,B>C†
PHQ-15-12	0.63 (±0.69)	0.50 (±0.65)	0.27 (±0.48)	0.000*	A,B>C†
PHQ-15-13	1.17 (±0.64)	0.95 (±0.72)	0.66 (±0.66)	0.000*	A>B>C
PHQ-15-14	0.87 (±0.76)	0.67 (±0.75)	0.54 (±0.70)	0.011*	A>C

A,B and C, for Groups A,B and C; The number after the questionnaire name indicates the number of the individual question in that questionnaire. **p*<0.05, †Analyzed by Games-Howell post hoc

Table **S4**. Depression with the PHQ-9 and anxiety with the GAD-7 scores in 309 patients.

	Mean (±SD)			*p*-value	Scheffe/ Games-Howell†
	Group A (n=70)	Group B (n=108)	Group C (n=131)		
PHQ-9 total score	7.77 (±5.71)	5.39 (±4.89)	3.69 (±3.92)	0.000*	A>B>C†
PHQ-9-1	1.11 (±1.00)	0.79 (±0.92)	0.56 (±0.83)	0.000*	A,B>C
PHQ-9-2	1.06 (±0.81)	0.79 (±0.89)	0.41(±0.70)	0.000*	A,B>C†
PHQ-9-3	1.26 (±0.99)	0.97(±1.09)	0.79(±0.93)	0.008*	A>C
PHQ-9-4	1.56 (±0.91)	1.14 (±1.02)	0.91 (±0.89)	0.000*	A,B>C
PHQ-9-5	0.86 (±1.00)	0.55 (±0.79)	0.43 (±0.74)	0.002*	A>C†
PHQ-9-6	0.51 (±0.83)	0.34 (±0.69)	0.15 (±0.49)	0.001*	A,B>C†
PHQ-9-7	0.66 (±0.92)	0.39 (±0.62)	0.11 (±0.36)	0.000*	A,B>C†
PHQ-9-9	0.26 (±0.65)	0.09 (±0.32)	0.06 (±0.30)	0.005*	-
GAD-7 total score	4.87 (±4.45)	3.61 (±3.79)	2.30 (±2.89)	0.000*	A,B>C†
GAD-7-1	0.86 (±0.95)	0.57 (±0.74)	0.29 (±0.56)	0.000*	A,B>C†
GAD-7-2	0.69 (±0.88)	0.50 (±0.73)	0.34 (±0.70)	0.007*	A>C†
GAD-7-3	1.09 (±0.88)	0.81 (±0.84)	0.58 (±0.75)	0.000*	A>B>C
GAD-7-5	0.30 (±0.62)	0.14 (±0.40)	0.05(±0.24)	0.000*	A>C
GAD-7-6	0.80 (±0.94)	0.71 (±0.85)	0.43 (±0.69)	0.003*	A,B>C†
GAD-7-7	0.37 (±0.71)	0.31 (±0.64)	0.13 (±0.36)	0.005*	A,B>C†

A,B and C, for Groups A,B and C; The number after the questionnaire name indicates the number of the individual question in that questionnaire. **p*<0.05, †Analyzed by Games-Howell post hoc

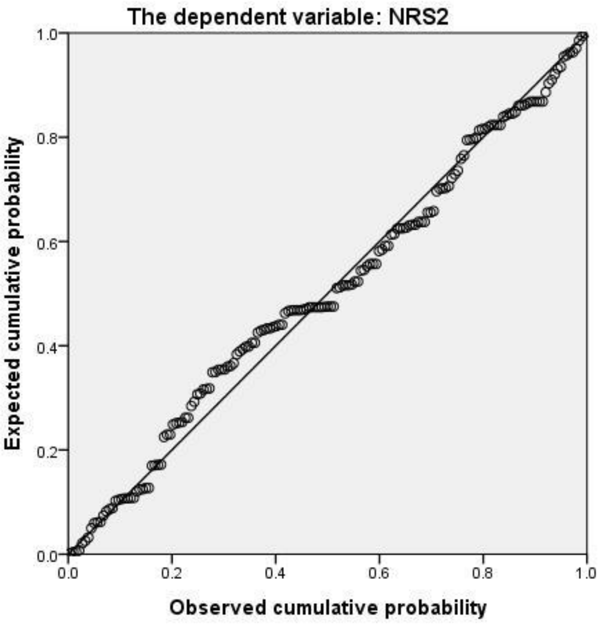

Figure S1. ZRESID Normal P-P Plot of multiple linear regression analysis in 171 patients.

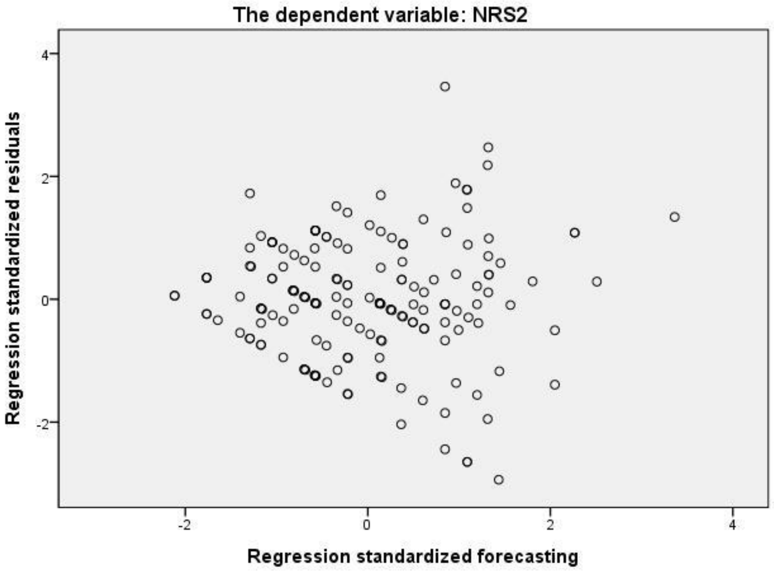

Figure S2. Scatterplot of ZRESID by ZPRED of multiple linear regression analysis in 171 patients.
